# Acquired Hemophilia

**DOI:** 10.4274/tjh.2014.0252

**Published:** 2014-12-05

**Authors:** Şinasi Özsoylu

**Affiliations:** 1 Retired Professor of Pediatrics, Hematology and Hepatology, Honorary Fellow of American Academy of Pediatrics, Honorary Member of American Pediatric Society

**Keywords:** Acquired hemophilia

## TO THE EDITOR

I would like to highlight 3 of our patients, a 14-year-old boy and females of 4 and 41 years old [1,2] with acquired hemophilia B seen at İhsan Doğramacı Children’s Hospital (previously Hacettepe) between 1963 and 1973 among 343 patients with hemophilia [3] on account of the case of acquired hemophilia A in a 78-year-old man who was successfully treated with a combined immunosuppressive and immunoadsorption approach by Bilgin et al. as reported in a recent issue of this journal [4].

Two of our 3 patients with acquired hemophilia B, which is rarer than acquired hemophilia A, improved most likely due to corticosteroid administration in at least one case and without any intervention in the others. We have shown antibodies against factor VIII in more than 20% of patients with X-linked hemophilia A and extremely rarely in normal people in low titers [3]; however, very severe bleeding due to the presence of AHG antibodies was not frequent.

On this occasion I would rather use the term ‘hereditary’ instead of ‘congenital’ hemophilia, which was used by Bilgin et al., since inheritance is not involved in most congenital disorders, such as congenital tuberculosis, syphilis, CMV, rubella infection, etc. May I also indicate that methyl prednisolone at 1 mg/kg/day may not be effective as an immunosuppressive, but 30 mg/kg/day seems to be more effective?

**Conflict of Interest Statement**

The authors of this paper have no conflicts of interest, including specific financial interests, relationships, and/or affiliations relevant to the subject matter or materials included.

## REPLY

I would like to thank you for your comment and interest in my article. Firstly you are right about this subject; hemophilia is a genetic disorder. However, congenital hemophilia is a term that can be used in literature. Secondly high dose of steroid was not preferred for this patient because of his age and comorbid disease.

**Aynur UĞUR BİLGİN**
